# Editorial: Epigenetic alterations in tumors and therapeutic resistance

**DOI:** 10.3389/fgene.2026.1863097

**Published:** 2026-05-08

**Authors:** Zhuoyi Peng, Cilu Huang, Jiangyuan Huang, Yue Chen, Shanzhou Huang

**Affiliations:** 1 Guangdong Provincial People’s Hospital (Guangdong Academy of Medical Sciences), Southern Medical University, Guangzhou, China; 2 Guangdong Cardiovascular Institute, Guangdong Provincial People’s Hospital (Guangdong Academy of Medical Sciences), Southern Medical University, Guangzhou, China

**Keywords:** biomarkers, cancer, epigenetic alterations, therapeutic resistance, tumors

## Introduction

Therapeutic resistance remains one of the principal barriers to improving clinical outcomes in oncology (Lei et al., 2020; Liu et al., 2020). Despite substantial advances in chemotherapy, targeted therapy, and immunotherapy over the past decade, both intrinsic and acquired resistance are pervasive and continue to compromise patient prognosis. Traditional paradigms have largely emphasized the role of genetic mutations in driving resistance. However, a growing body of evidence indicates that epigenetic alterations, which are heritable and reversible changes in gene expression that occur without modification of the DNA sequence, play a pivotal role in the initiation and progression of therapeutic resistance (Wajapeyee and Gupta, 2021; Song et al., 2025).

The present thematic collection, “Epigenetic Alterations in Tumors and Therapeutic Resistance,” brings together a series of cutting-edge studies that systematically interrogate the multifaceted contributions of epigenetic mechanisms to resistance development. Spanning RNA modifications, immune regulation, and tumor microenvironment remodeling, these studies provide an integrated perspective on the epigenetic landscape of therapy resistance. Unlike previous studies that mainly focused on DNA methylation and histone modifications, this topic highlights the integrative role of RNA-level epigenetic regulation in therapeutic resistance and its close association with tumor immunity.

## Central regulatory role of RNA epigenetic modifications in therapeutic resistance

In recent years, epitranscriptomics has rapidly advanced. RNA modifications, particularly m6A methylation, have been recognized as key mechanisms in cancer therapeutic resistance (Huang et al., 2020; Liu et al., 2022; Zhuang et al., 2023). Studies included in this topic, focusing on multiple tumor types such as gastric cancer and osteosarcoma, demonstrate that m6A modification regulates mRNA stability, splicing, and translation efficiency (Yang et al.; Zhou et al.). Through these mechanisms, it is widely involved in critical biological processes, including drug metabolism, cell cycle regulation, and DNA damage repair, thereby directly influencing treatment sensitivity. Furthermore, the roles of key “writer” enzymes, such as RBM15 and METTL14, in tumor progression and immune regulation are systematically characterized (Li et al.; Li et al.). These factors not only reshape gene expression programs at the post-transcriptional level but also establish important links between RNA modification and tumor immunity by regulating immune-related molecule expression and immune cell infiltration. In addition, studies on pseudouridine-modifying enzymes further expand the scope of RNA epigenetic regulation, suggesting that different types of RNA modifications may act synergistically to mediate adaptive reprogramming of tumors under therapeutic pressure (Liu et al.). Meanwhile, studies in cervical cancer show that long non-coding RNAs regulate miRNA activity and key pathways such as PI3K/AKT and Wnt/β-catenin through ceRNA networks, contributing to resistance to chemotherapy and radiotherapy (He et al.). Collectively, these findings indicate that RNA-level epigenetic regulation has evolved from a supportive role to one of the central mechanisms driving therapeutic resistance (Song et al., 2020; Uddin et al., 2024).

## Epigenetically driven tumor immune evasion

Immunotherapy, particularly the use of immune checkpoint inhibitors, has significantly improved clinical outcomes in multiple cancers (Wei et al., 2024; Bagchi et al., 2021). However, immune resistance remains a common challenge. Several reviews in this collection systematically examine the mechanisms of immune evasion from an epigenetic perspective. Evidence shows that epigenetic alterations can suppress anti-tumor immune responses through multiple pathways, including reducing tumor antigen expression, inhibiting antigen presentation, upregulating immune checkpoint molecules, and promoting the recruitment of immunosuppressive cells. In studies on glioma, it is reported that DNA methylation and histone modifications can jointly regulate the expression of immune-related genes, leading to the formation of an immunosuppressive microenvironment and reduced efficacy of immunotherapy (Wu et al.). In hepatocellular carcinoma, further research reveals that CA19-9-related signaling can drive the polarization of tumor-associated macrophages toward an immunosuppressive phenotype, thereby decreasing the effectiveness of immune checkpoint inhibitors (Du et al.). These findings highlight that epigenetic regulation not only affects tumor cells directly but also promotes therapeutic resistance by reshaping the tumor immune microenvironment (Cao and Yan, 2020; Yang et al., 2023).

## Epigenetic remodeling in the tumor microenvironment and therapeutic resistance

Accumulating evidence suggests that therapeutic resistance is not driven by a single mutational event but represents a dynamic adaptive process (França et al., 2024; Vander et al., 2020). Studies in this collection support the view that tumor cells can rapidly reprogram their transcriptional states through reversible epigenetic alterations under therapeutic pressure, thereby acquiring transient or stable resistant phenotypes. During this process, RNA modifications, lncRNA regulation, and changes in the immune microenvironment interact to form a complex network that drives resistance. In solid tumors such as oral squamous cell carcinoma, epigenetic regulation has been confirmed to further promote tumor progression and treatment resistance by influencing the activation of tumor-associated fibroblasts, inflammatory signals and extracellular matrix remodeling (Li et al.). This high degree of plasticity not only explains the presence of drug-tolerant persister cells but also provides a theoretical basis for reversing therapeutic resistance (He et al., 2024; Russo et al., 2024).

## Epigenetic therapeutic strategies and their potential in combination approaches

Because epigenetic alterations are reversible, targeting epigenetic regulators has emerged as an important strategy to overcome therapeutic resistance (Wang et al., 2023). Studies in this collection consistently show that single-agent epigenetic therapies have limited efficacy, whereas combination strategies are more promising. lncRNA networks or Targeting m6A regulators such as METTL14 and RBM15 may help restore tumor sensitivity to chemotherapy, targeted therapy, and immunotherapy. In addition, epigenetic modulation can enhance antigen expression and improve the immune microenvironment, thereby increasing the efficacy of immune checkpoint inhibitors. With advances in multi-omics technologies, precision treatment strategies based on individual epigenetic profiles are becoming increasingly feasible.

## Challenges and future directions

Although epigenetic research has provided new insights into tumor therapeutic resistance, several challenges remain. First, epigenetic regulatory networks are highly complex, with extensive crosstalk among different mechanisms, which increases the difficulty of investigation (Zhao et al., 2021). Second, the lack of stable and reliable epigenetic biomarkers limits their clinical application. In addition, both inter-tumor and intra-tumor heterogeneity pose major challenges to precision therapy (Dagogo-Jack and Shaw, 2018). Future research should focus on several key directions, including multi-omics approaches integrating genomics, epigenomics, and transcriptomics; single-cell and spatial omics for resolving tumor heterogeneity; and the dynamic monitoring of epigenetic changes over time. In addition, efforts should be directed toward developing highly specific and low-toxicity epigenetic-targeted agents, as well as optimizing combination therapies to achieve personalized precision treatment.

Therefore, these studies collectively support a new integrative framework in which therapeutic resistance is not a single molecular event, but a dynamic adaptive process driven by epigenetic reprogramming at the RNA level. This process is mediated through the coordinated actions of non-coding RNA regulation, RNA chemical modifications, and remodeling of the immune microenvironment. This perspective not only deepens our understanding of resistance mechanisms, but also suggests that future strategies should focus on RNA epigenetic regulation as a central target, with combined immunotherapy and targeted therapy approaches to achieve precise intervention in tumor resistance.
